# Long noncoding RNA GAS5 inhibits cell proliferation and fibrosis in diabetic nephropathy by sponging miR-221 and modulating SIRT1 expression

**DOI:** 10.18632/aging.102249

**Published:** 2019-10-20

**Authors:** Xiaoxu Ge, Bojin Xu, Wenwei Xu, Lili Xia, Zhongqin Xu, Lisha Shen, Wenfang Peng, Shan Huang

**Affiliations:** 1 Department of Endocrinology, Tongren Hospital Affiliated to Shanghai Jiaotong University, Shanghai, China; 2 Department of Geriatrics, Tongren Hospital Affiliated to Shanghai Jiaotong University, Shanghai, China; 3 Department of Family Medicine, Tongren Hospital Affiliated to Shanghai Jiaotong University, Shanghai, China

**Keywords:** diabetic nephropathy, proliferation, fibrosis, lncRNA GAS5

## Abstract

Diabetic nephropathy (DN) is one of the leading causes of end-stage renal diseases worldwide. This study is designed to investigate the underlying function and mechanism of a novel lncRNA GAS5 in the progression of DN. We found that lncRNA GAS5 expression level was decreased in type 2 diabetes (T2D) with DN compared with that in patients without DN. Moreover, lncRNA GAS5 expression level was negatively associated with the severity of DN-related complications. lncRNA GAS5 inhibited MCs proliferation and caused G0/1 phase arrest. lncRNA GAS5 overexpression alleviated the expression of fibrosis-related protein in mesangial cells (MCs). The dual-luciferase reporter assay and RNA binding protein immunoprecipitation (RIP) assay results revealed that lncRNA GAS5 functions as an endogenous sponge for miR-221 via both the directly targeting way and Ago2-dependent manner. Furthermore, SIRT1 was confirmed as a target gene of miR-221. lncRNA GAS5 upregulated SIRT1 expression and inhibited MCs proliferation and fibrosis by acting as an miR-221 sponge. Finally, we found that lncRNA GSA5 suppressed the development of DN in vivo. Thus, lncRNA GAS5 was involved in the progression of DN by sponging miR-221 and contributed to lncRNA-directed diagnostics and therapeutics in DN.

## INTRODUCTION

Diabetic nephropathy (DN) poses considerable threat to public health [1, 2]. DN is one of the main complications of diabetes mellitus and the primary cause of renal failure [3]. Despite the increasing number of therapeutic methods to treat the disease, the incidence rate of DN continues to increase [4, 5]. Thus, an understanding of the underlying mechanism of progression of DN is urgently needed.

Long noncoding RNAs (lncRNAs) constitute a large class of 200nt-long noncoding transcripts that lack protein-coding capacity [6]. A large number of studies have revealed that lncRNAs play a vital role in the development of DN. lncRNA MIAT upregulates the TGFβ1 signaling pathway and enhances diabetic retinopathy [7]. In diabetic mice, the downregulation of lncRNA H19 suppresses the abnormal differentiation of small intestinal epithelial cells by competing with miR-141-3p [8]. The knockdown of HOTTIP regulates the cell cycle and insulin secretion by suppressing MEK/ERK axes in islet β cells [9]. Growth arrest-specific 5 (GAS5, ID: 60674) is a novel marker in diabetes mellitus. Decrease in circulating lncRNA GAS5 is positively associated with diabetes and offered a new tool for identifying people at risk of diabetes [10]. Additionally, lncRNA GAS5 acts as an anti-oncogene in various tumors. lncRNA GAS5 inhibits invasion, epithelial-mesenchymal transition (EMT), proliferation, and invasion in oral squamous cell carcinoma by modulating the miR-21/PTEN pathway [11]. The knockdown of lncRNA GAS5 enhances the development of ovarian cancer by sponging miR-196-5p and modulating HOXA5 expression [12]. However, the biological function and potential mechanism of lncRNA GAS5 in DN are not fully understood.

MicroRNAs (miRNAs) are 20–25 nucleotides in length and regulate coding genes though post-transcriptional modulation [13]. Competing endogenous RNA (ceRNA) is a vital regulatory mechanism between lncRNAs and miRNAs and has been identified as a novel mechanism in DN. lncRNA-NR_033515 enhances EMT, fibrogenesis and proliferation by sponging miR-743b-5p in DN [14]. LincRNA 1700020I14Rik inhibits cell fibrosis and proliferation by sponging miR-34a-5p and activating Sirt1/HIF-1α signaling [15]. LncRNA LINC01619 promotes endoplasmic reticulum stress-mediated podocyte injury by acting as a sponge for miR-27a in DN [16]. Furthermore, miR-221 is involved in the progression of diabetes. Serum miR-221 serves as a novel biomarker for patients with diabetic retinopathy [17]. The overexpression of miR-221 enhances neointimal hyperplasia in diabetes mellitus by kinases ½ activity [18].

In the present study, we found that lncRNA GAS5 was downregulated in DN in vivo and in vitro. Moreover, lncRNA GAS5 regulated proliferation and fibrosis in MCs, upregulated SIRT1 expression, and inhibited cell proliferation and fibrosis by acting as an miR-221 sponge. Thus, this study revealed the underlying mechanism for GAS5 in the progression of DN and provided a potential target for the prevention or treatment of DN.

## RESULTS

### LncRNA GAS5 was downregulated in DN and negatively associated with the severity of DN-related complications

**Figure 1:**
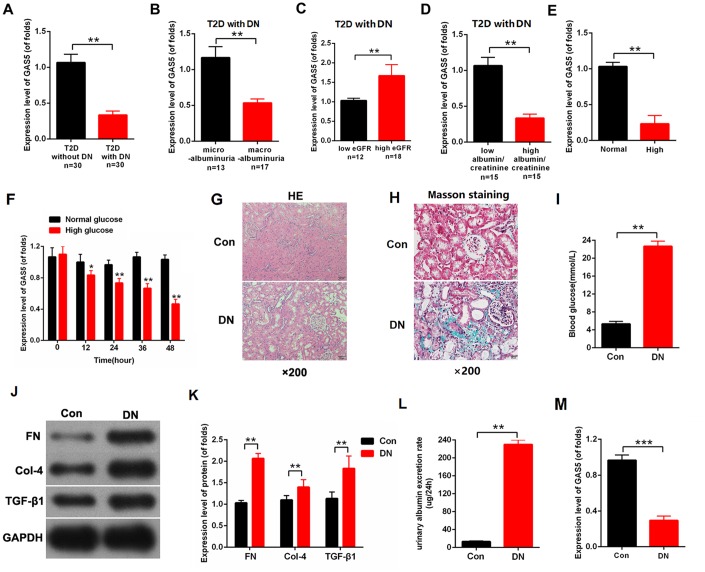
**lncRNA GAS5 was downregulated in DN and negatively associated with the severity of DN-related complications.** (**A**) Expression levels of GAS5 was detected by qPCR in T2D with or without DN (n=30); (**B**) Expression levels of GAS5 was detected by qPCR in DN with microalbuminuria or with macroalbuminuria (microalbuminuria group=13, macroalbuminuria group=17); (**C**) Based on the average value of eGFR, expression levels of GAS5 were detected in low or high eGFR (low eGFR group=12, high eGFR group=18); (**D**) Based on the average value of albumin/creatinine, expression levels of GAS5 were detected in low or high albumin/creatinine (low albumin/creatinine=15, high albumin/creatinine=15); (**E**) Expression levels of GAS5 were detected by qPCR in MCs treated with high glucose and compared with those in MC treated with normal glucose after 36 h treatment; (**F**) Expression levels of GAS5 were detected by qPCR in MCs treated by high glucose for 0, 4, 8 day; (**G**) H&E staining results of the DN rat model and negative control. Results indicated that the epithelium cells of tubule were edematous. Glomerular mesangial proliferation is shown in DN (N=8); (**H**) Blood glucose was detected in the DN rat model and negative control (N=8); (**I**, **J**) fibrosis-related proteins (FN, Col-4, and TGF-β) were measured in the DN rat model and negative control (N=8); (**K**) Urinary albumin excretion rate was measured in the DN rat model and negative control (N=8); (**L**) Expression level of GAS5 was detected in the DN rat model and negative control (**M**) The expression level of GAS5 was detected by qPCR in the DN rate model and negative control. Results showed that the expression level of GAS5 was downregulated in the DN rat model (N=8). Three independent experiments were performed. *P < 0.05, **P < 0.01.

To investigate the relationship between lncRNA GAS5 and DN, we collected the kidney tissues of patients with type 2 diabetes (T2D) (T2D with DN=30, T2D without DN=30). The clinical characteristics of patients are provided in Table 1. As shown in Figure 1A, the expression level of lncRNA GAS5 decreased in T2D with DN compared with that in T2D without DN. In T2D with DN, lncRNA GAS5 was negatively associated with the severity of DN-related complications. Specifically, the expression level of GAS5 was decreased in macro albuminuria compared with micro albuminuria (Figure 1B). Based on the average value of estimated glomerular filtration rate (eGFR), patients with DN were divided into low eGFR group (N=12) and high eGFR group (N=18). qPCR results indicated that the expression level of GAS5 was reduced in the low eGFR group compared with that of the high eGFR group (Figure 1C). Then, the expression level of GAS5 was detected in low albumin/creatinine or high albumin/creatinine level defined by the average value of albumin/creatinine. As shown in Figure 1D, the expression level of GAS5 was reduced in the high albumin/creatinine group. Moreover, the mesangial cells (MCs) were treated by normal or high glucose (NG (normal glucose) =5.5 mmol/L glucose, HG (high glucose) =25 mmol/L glucose). The expression level of lncRNA GAS5 decreased in MCs treated with high glucose (Figure 1E). The expression level of lncRNA GAS5 was detected across time. The expression level of lncRNA GAS5 treated with high glucose gradually declined with time (Figure 1F). To further study the expression pattern of lncRNA GAS5 in vivo, we established DN rat models. The hematoxylin-eosin staining (H&E staining) results of the DN rat model and negative control indicated that the epithelium cells of tubule were edematous, and glomerular mesangial proliferation was observed in DN (N=8) (Figure 1G). Moreover, Masson staining showed that there were massive deposits of collagen (blue color) in the DN model (Figure 1H). To further verify the success of the model construction, we detected the blood glucose, fibrosis-related proteins, and urinary albumin excretion rate of the DN rat model. The results showed that blood glucose (Figure 1I), fibrosis-related proteins (Figure 1J, 1K), and urinary albumin excretion rate (Figure 1L) were significantly increased in the DN rat model compared with those of the NC group. Finally, the expression level of lncRNA GAS5 was detected. The qPCR results showed that the expression level of lncRNA GAS5 was significantly decreased in the DN rat model (Figure 1M).

**Table 1:** Clinical characteristics of type 2 diabetic patients without or with DN (N=60).

**Variable**	**Type 2 diabetes without DN**	**Type 2 diabetes with DN**	**P value**
Age	50±4	52±3	NS
N(% male)	36(60%)	40(66.7%)	NS
BMI(kg/m2)	83±10.1	85±12.6	NS
Glucose (mg/dl)	151±22.3	155±17.3	NS
HbA1c(%)(mmol/mol)	7.41±1.5	7.55±1.6	NS

### LncRNA GAS5 inhibited proliferation in MCs

**Figure 2:**
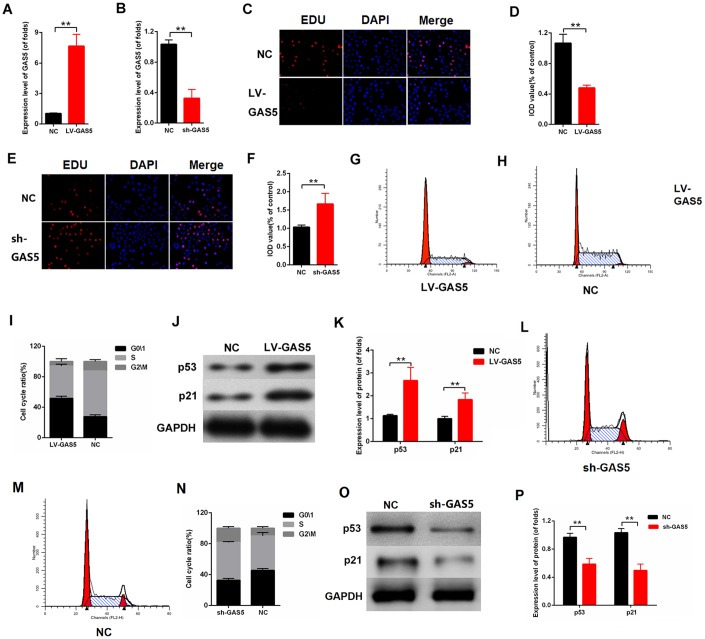
**lncRNA GAS5 alleviated MC proliferation.** (**A**–**B**) Efficiency of LV-GAS5 and sh-GAS5 were detected by qPCR; (**C**-**D**) Proliferating mesangial cells were labeled with EdU. MCs were transfected with LV-GAS5; (**E**–**F**) Proliferating mesangial cells were labeled with EdU. MCs were transfected with sh-GAS5; (**G**–**I**) Flow cytometric assay showed that GAS5 increased the G0/1 phase of MCs. MCs were transfected with LV-GAS5; (**J**–**K**) p53 and p21 expression levels were measured by western blot. MCs were transfected with LV-GAS5. *P < 0.05, **P < 0.01.

To further investigate the effect of lncRNA GAS5 on cell proliferation, lncRNA GAS5 overexpression or knockdown was carried out. The efficiency of LV-GAS5 and sh-GAS5 was detected by qPCR (Figure 2A, 2B). As indicated in Figure 2C, 2D, the upregulation of lncRNA GAS5 inhibited proliferation in MCs, whereas the downregulation of lncRNA GAS5 enhanced proliferation (Figure 2E, 2F). Flow cytometry analysis was performed, and the results revealed that MCs was arrested in the G0/1phase after they were transfected with LV-GAS5 (Figure 2F–2I). The Western blot results revealed that lncRNA GAS5 overexpression increased the expression level of p53 and p21 (Figure 2J, 2K). Overall, our findings demonstrated that lncRNA GAS5 inhibits cell proliferation in MCs and increases p53 and p21 expression.

### LncRNA GAS5 suppressed the expression level of fibrosis-related proteins in MCs

**Figure 3:**
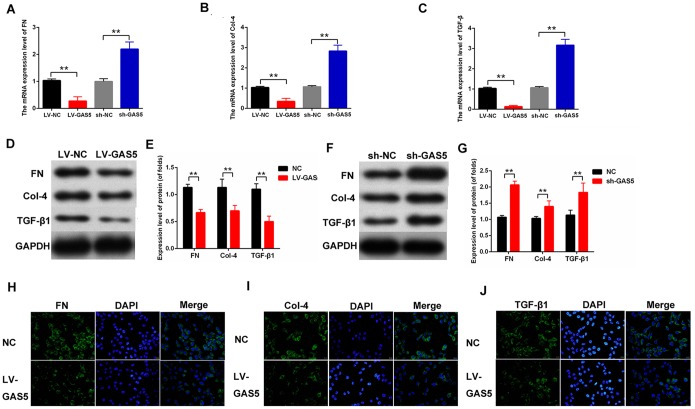
**lncRNA GAS5 downregulated the expression level of fibrosis-related proteins in MCs.** (**A**–**C**) mRNA expression levels of FN (**A**), Col-4 (**B**), and TGFβ1 (**C**) in MCs were measured by qPCR. MCs were transfected with LV-GAS5 or sh-GAS5; (**D**–**G**) FN, Col-4, and TGFβ1 expression levels were detected by western blot. MCs were transfected with LV-GAS5 and sh-GAS5; (**H**–**J**) immunofluorescence analysis showed that FN, Col-4 and TGFβ1 expression levels were downregulated. MCs were transfected with LV-GAS5. *P < 0.05, **P < 0.01.

Fibrosis changes are the pathologic bases of DN. MCs are the major participants in the development of fibrosis. To study the effects of lncRNA GAS5 on the fibrosis of MCs, DN fibrosis-related proteins were detected. The qPCR results revealed that LV-GAS5 inhibited the mRNA expression level of fibrosis- related proteins and sh-GAS5 had an opposite effect on fibrosis-related proteins, including FN, Col-4, and TGFβ1 (Figure 3A–3C). The protein expression levels of the fibrosis-related proteins were then detected. The Western blot results showed that LV-GAS5 suppressed the expression of FN, Col-4, and TGFβ1, and sh-GAS5 enhanced the expression of FN, Col-4, and TGFβ1 (Figure 3D–3G). Furthermore, the immunofluorescence assay revealed that the expression levels of FN, Col-4, and TGFβ1 were reduced in MCs transfected with LV-GAS5 (Figure 3H–3J). Overall, these data suggested that lncRNA GAS5 alleviates fibrosis-related proteins in MCs.

### LncRNA GAS5 sponged miR-221 through both directly targeting and Ago2-dependent manner

**Figure 4:**
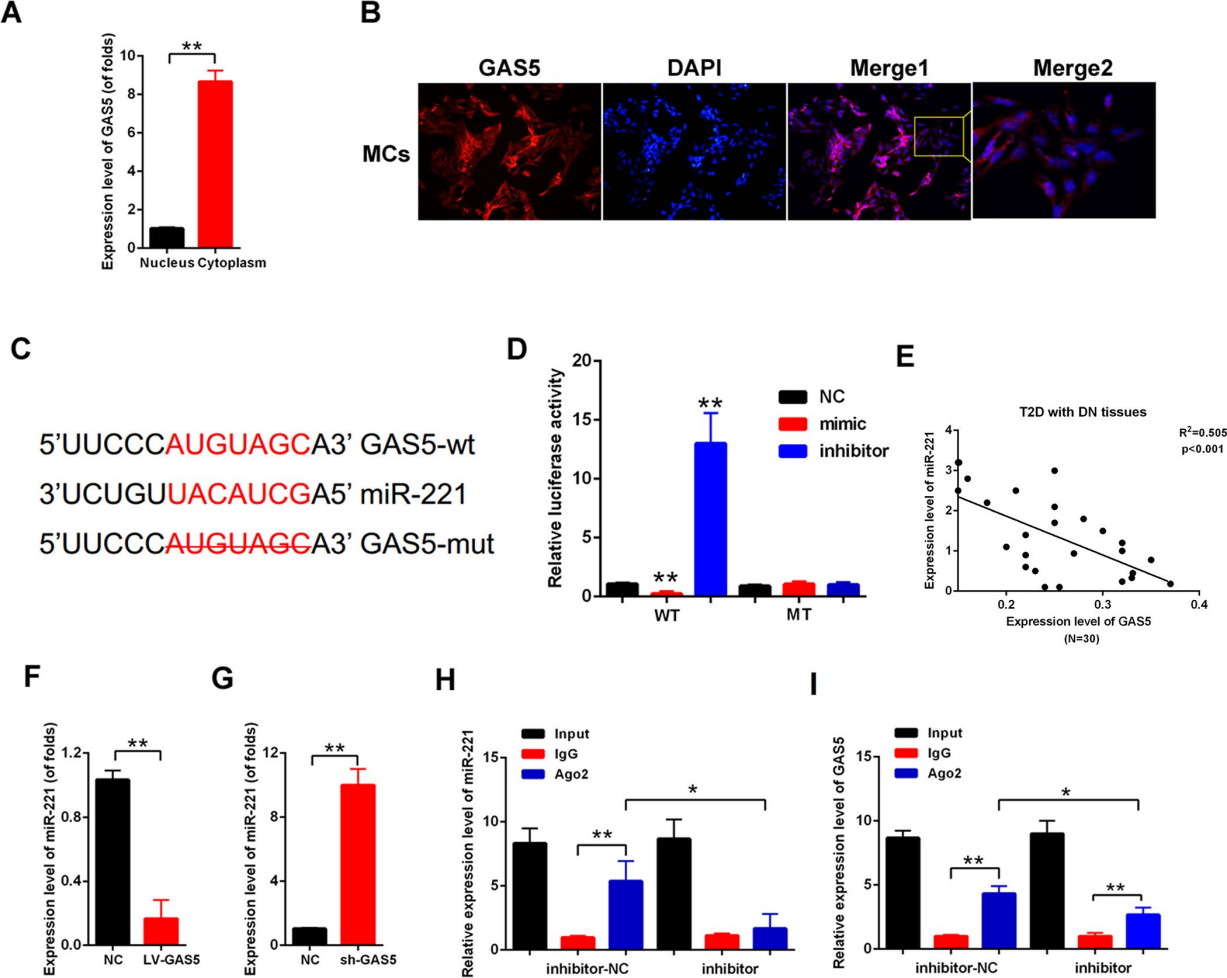
**lncRNA GAS5 sponged miR-221 through both directly targeting and Ago2-dependent manner.** (**A**) qPCR results showed that GAS5 expression level in nucleus was higher than that in the cytoplasm; (**B**) FISH results indicated that GAS5 was chiefly located in the cytoplasm; (**C**) Direct binding sites between GAS5 and miR-221 were revealed; (**D**) Binding relationship between GAS5 and miR-221 was detected by luciferase reporter assay with wild or mutant type of luciferase reporter plasmids of GAS5; (**E**) There is negative correlation between lncRNA GAS5 and miR-221 in T2D with DN tissues (N=30). (**F**, **G**) miR-221 expression was detected by qPCR. MCs were transfected with LV-GAS5 and sh-GAS5; (**H**, **I**) RIP assay was performed, which used input from cell lysate, IgG, or anti-Ago2. The expression levels of GAS5 and miR-221 were measured by qPCR.

Competing endogenous RNAs (ceRNAs) have become vital regulatory mechanisms for lncRNAs [19]. First, acting as an effective miRNA sponge, lncRNA should be mainly expressed in the cytoplasm. Nuclear and cytoplasmic RNA extraction was performed. The qPCR results showed that lncRNA GAS5 was expressed higher in cytoplasm than in the nucleus (Figure 4A). Moreover, FISH images showed that lncRNA GAS5 was mainly located in the cytoplasm of the MCs (Figure 4B). The potential target miRNAs of lncRNA GAS5 were predicted by LncBase (https://diana.e-ce.uth.gr/lncbasev3). MiR-221 was predicted as a candidate of lncRNA GAS5. The direct binding between lncRNA GAS5 and miR-221 is shown in Figure 4C. Luciferase reporter assay was carried out to confirm the direct binding relationship between lncRNA GAS5 and miR-221. The results showed that lncRNA GAS5 wild-type reporter gene was decreased in luciferase activity by miR-221. However, the mutant-type reporter gene was not inhibited in luciferase activity by miR-221 (Figure 4D). Moreover, the relationship between lncRNA GAS5 and miR-221 was detected in T2D with DN tissues (N=30). Result showed that there is negative correlation between lncRNA GAS5 and miR-221 (Figure 4E). Then, qPCR results showed that lncRNA GAS5 overexpression decreased miR-221 expression, whereas lncRNA knockout increased miR-221 expression in the MCs (Figure 4F, 4G). miRNAs serve as part of RNA-induced silencing complex (RISC), and lncRNA regulates gene expression though an RISC [15]. We carried out RIP analysis to investigate whether lncRNA GAS5 and miR-221 are in an RISC. As indicated in Figure 4H, 4I, the expression levels of lncRNA GAS5 and miR-221 increased in the anti-Ago2 group compared with those of the control group. In the anti-miR-221 group, the expression levels of lncRNA GAS5 and miR-221 immunoprecipitated with Ago2 decreased compared with those of the anti-normal IgG group. These results revealed that lncRNA GAS5 decrease miR-221 expression in the manner of directly targeting and Ago2-dependent manner.

### MiR-221 inhibition attenuated proliferation and fibrosis-related proteins

**Figure 5:**
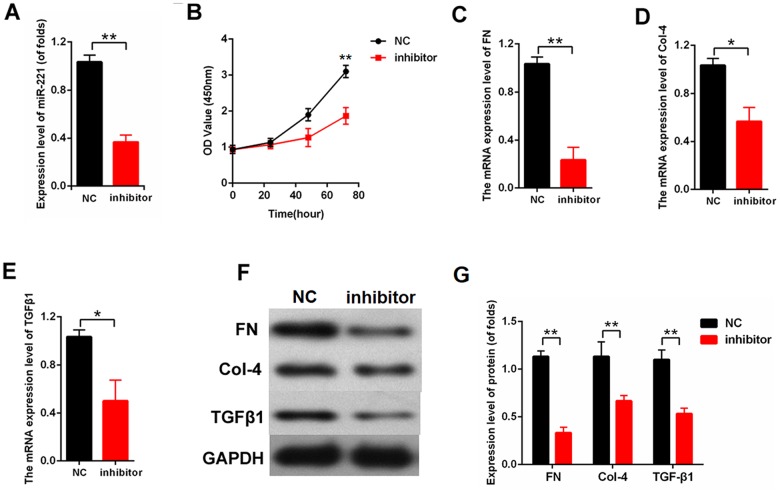
**Downregulation of miR-221 attenuated proliferation and fibrosis-related proteins.** (**A**) The efficiency of inhibitor-miR-221 was measured by qPCR; (**B**) CCK8 assay results showed that miR-221 inhibition suppressed MC proliferation; (**C**–**E**) mRNA expression level of FN (**C**), Col-4 (**D**), and TGFβ1 (**E**) in MCs transfected with inhibitor-miR-221; (**F**, **G**) The protein expression level of FN, Col-4, and TGF-β1 were measured by western blot. MCs were transfected with inhibitor-miR-221. *P < 0.05, **P < 0.01.

We knocked out miR-221 to further investigate the effect of miR-221 on proliferation and fibrosis. The efficiency of inhibitor-miR-221 was measured by qPCR (Figure 5A). miR-221 inhibition significantly decreased cell proliferation in the MCs detected by CCK8 (Figure 5B). Moreover, the expression levels of FN, Col-4, and TGFβ1 were detected. The qPCR results revealed that miR-221 inhibition downregulated the mRNA expression levels of FN, Col-4, and TGFβ1 in the MCs (Figure 5C–5E). Furthermore, our finding showed that miR-221 inhibition decreased the protein expression levels of FN, Col-4, and TGFβ1 in the MCs (Figure 5F, 5G). Therefore, these data indicated that miR-221 inhibition attenuated proliferation and fibrosis-related proteins in MCs.

### MiR-221 inhibited the proliferation and fibrosis-related proteins by targeting SIRT1

**Figure 6:**
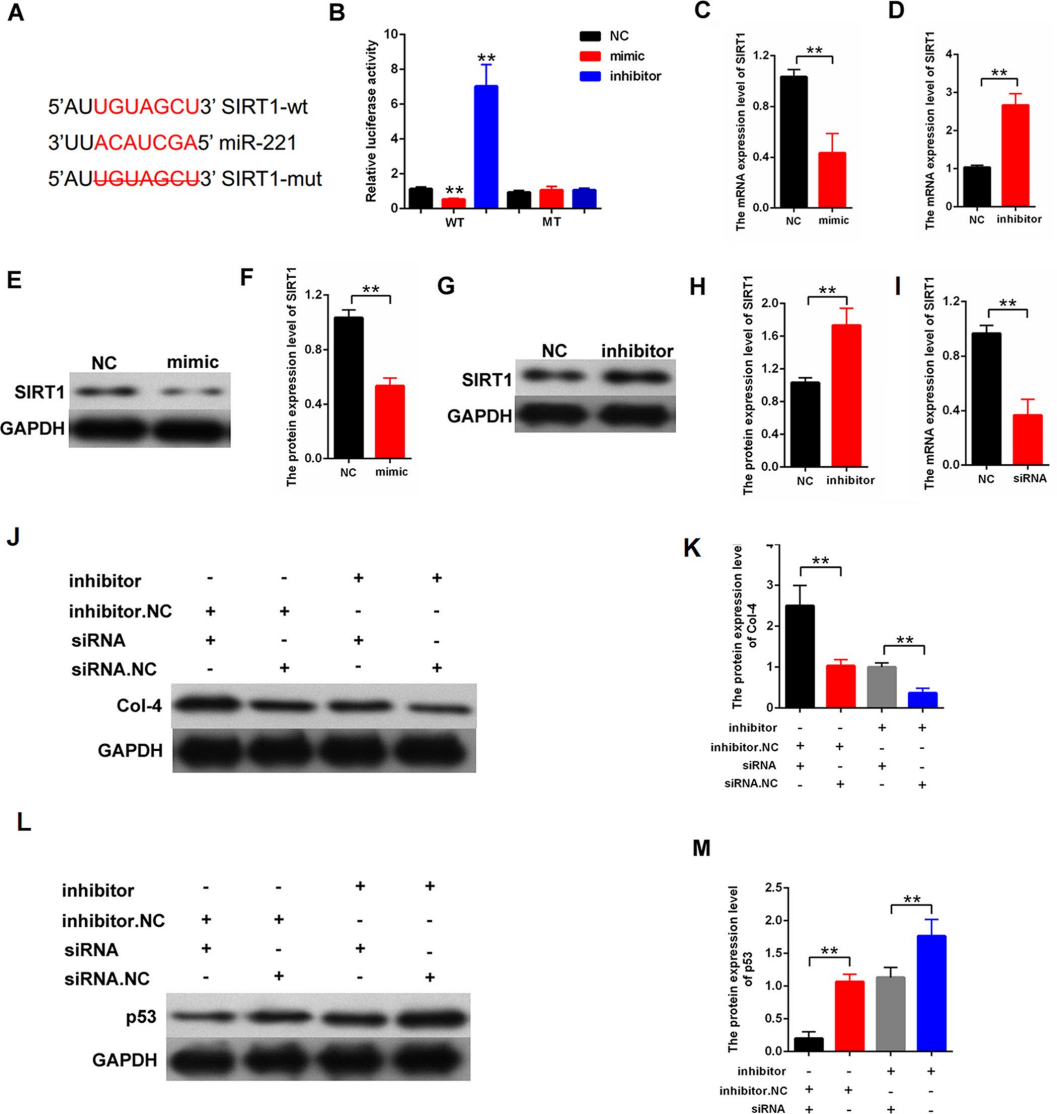
**miR-221 inhibited proliferation and fibrosis-related proteins by targeting SIRT1.** (**A**) Directly binding region between miR-221 and SIRT1; (**B**) Binding relationship between miR-221 and SIRT1 was detected by luciferase reporter assay with wild or with the mutant type of the luciferase reporter plasmids of SIRT1; (**C**–**H**) qPCR and western blot assays revealed that mimic-miR-221 inhibited SIRT1 expression, and inhibitor-miR-221 enhanced SIRT1 expression; (**I**) The efficiency of siRNA-SIRT1 was measured in MCs by qPCR; (**J**–**K**) Protein expression level of Col-4 was measured by western blot in the MCs, which were transfected with inhibitor-miR-221 or siRNA-SIRT1; (**L**–**M**) protein expression level of p53 was measured by western blot in the MCs, which were transfected with inhibitor-miR-221 or siRNA-SIRT1. *P < 0.05, **P < 0.01.

The candidate target genes of miR-221 were predicted by the miRNA.org (http://www.microrna.org/microrna/home.do). SIRT1 was predicted as a candidate gene of miIR-221. The direct binding between miR-221 and SIRT1 is shown in Figure 6A. Luciferase reporter assay indicated that SIRT 3′UTR wild-type reporter gene was decreased in luciferase activity by miR-221. However, the 3′UTR mutant-type reporter gene was not decreased in luciferase activity by miR-221 (Figure 6B). Then, qPCR and Western blot results indicated that miR-221-mimic decreased SIRT1 expression, and miR-221-inhibitor increased SIRT1 expression in the MCs (Figure 6C–6H). Furthermore, the role of SIRT1 in miR-221 function was also explored. A rescuing experiment was carried out in the MCs. The results revealed that silencing SIRT1 restores the negative regulation effect of inhibitor-miR-221 on proliferation and fibrosis in MCs. The cells were transfected with inhibitor-miR-221 or siRNA-SIRT1. On the one hand, SIRT1 silencing can restore the significant inhibition of Col-4 expression after transfection with inhibition-miR-221. On the other hand, SIRT1 silencing can restore the significant improvement of p53 expression after transfection with inhibition-miR-221. (Figure 6G, 6H). Thus, our finding indicated that miR-221 inhibited proliferation and fibrosis by targeting SIRT1.

### LncRNA GAS5 upregulated SIRT1 expression and attenuated proliferation and fibrosis-related proteins by acting as an miR-221 sponge

**Figure 7:**
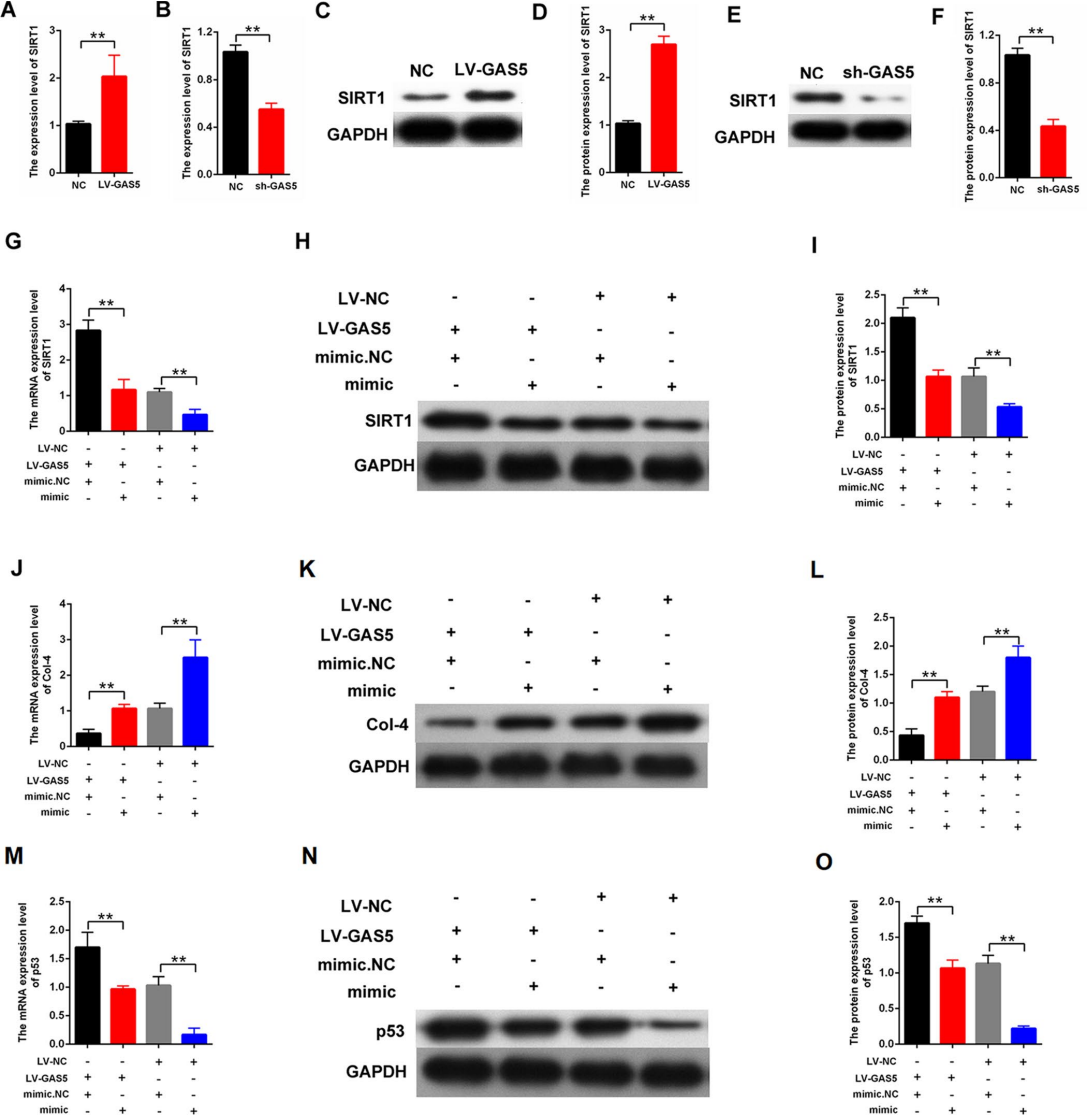
**lncRNA GAS5 upregulated SIRT1 expression and decreased the proliferation and fibrosis-related proteins by acting as miR-221 sponge.** (**A**–**F**) qPCR and western blot assays revealed that GAS5 upregulation increased SIRT1 expression and GAS5 downregulation decreased SIRT1 expression; (**G**–**I**) qPCR and western blot results showed that the expression level of SIRT1 in the MCs. The MCs were transfected with LV-GAS5 or mimic-miR-221; (**J**–**L**) expression level of Col-4 in MCs was measured by qPCR and western blot. The MCs were transfected with LV-GAS5 or mimic-miR-221; (**M**–**O**) p53 expression level in MCs was measured by qPCR and western blot. MCs were transfected with LV-GAS5 or mimic-miR-221. *P < 0.05, **P < 0.01.

We performed qPCR and western blot analysis to investigate the relationship between lncRNA GAS5 and SIRT1. Specifically, lncRNA GAS5 overexpression enhanced the expression level of SIRT1, and lncRNA GAS5 silencing attenuated SIRT1 expression (Figure 7A–7F). Furthermore, the role of miR-221 in lncRNA GAS5 function was explored. MCs were transfected with LV-GAS5 or mimic-221. The rescuing experiment further revealed that mimic-miR-221 can restore the upregulation of SIRT1 after transfection with LV-GAS5 (Figure 7G–7I). Moreover, the effect of miR-221 on MC proliferation and fibrosis was investigated. Specifically, qPCR and western blot indicated that mimic-miR-221 can restore the downregulation of Col-4 after transfection with LV-GAS5 (Figure 7J–7L). Additionally, mimic-miR-221 restored the upregulation of p53 after transfection with LV-GAS5 (Figure 7M–7O). Overall, lncRNA GAS5 enhanced the expression level of SIRT1 and inhibited MC proliferation and fibrosis by targeting miR-221.

### Upregulation of lncRNA GAS5 alleviated DN in vivo

**Figure 8:**
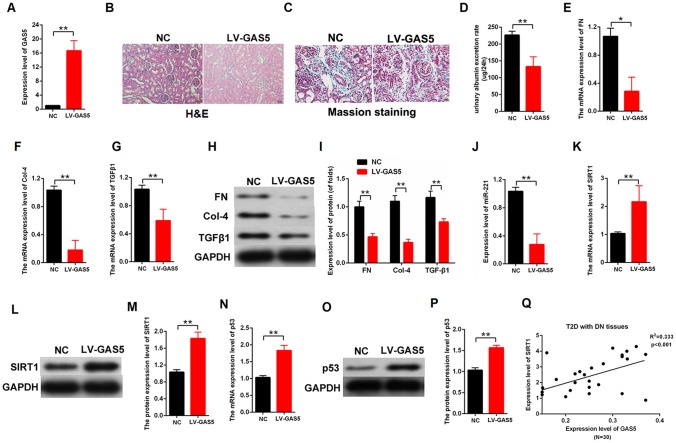
**Upregulation of lncRNA GAS5 alleviated DN in vivo.** (**A**) H&E staining of DN results. The LV-GAS5 group was injected with LV-GAS5, and the LV-NC group was injected with LV-NC (LV-GAS5 group=8, LV-NC group=8); (**B**) The expression level of lncRNA GAS5 was measured by qPCR in both groups; (**C**) Urinary albumin excretion rate was decreased in the LV-GAS5 group compared with that in the LV-NC group; (**D**–**F**) The expression level of FN (**D**), Col-4 (**E**), and TGFβ1 (**F**) were measured by qPCR in the DN rat model transfected with LV-GAS5 or LV-NC; (**G**–**H**) The expression level of FN, Col-4, and TGFβ1 were measured by western blot in the DN rat model transfected with LV-GAS5 or LV-NC; (**I**) qPCR results revealed that the expression level of miR-221 decreased in the DN rat model transfected with LV-GAS5; (**J**–**L**) The expression level of SIRT1 was upregulated in the DN rat model transfected with LV-GAS5; (**M**–**O**) The expression level of p53 was upregulated in DN transfected with LV-GAS5; (**P**) qPCR results revealed that there is positively correlation between lncRNA GAS5 and SIRT1 (Figure 8P). (**Q**) The relationship between the expression level of GAS5 and SIRT1 was detected by qPCR in T2D with DN tissues (N=30). *P < 0.05, **P < 0.01.

We established DN rat models to confirm the lncRNA GAS5 effect in vivo. The LV-GAS5 group (N=8) and LV-NC group (N=8) were established by injection with LV-GAS5 and LV-NC, respectively. The qPCR results showed that LV-GAS5 overexpression in the DN rat models was successfully established (Figure 8A). The H&E stains of the DN results were observed with a microscope (Figure 8B). Moreover, Masson staining revealed that there were less deposits of collagen (blue color) in the DN model transfected with LV-GAS5 compared with negative control (Figure 8C). We found that lncRNA GAS5 overexpression alleviated urinary albumin excretion rate in the DN rat models (Figure 8D). Total proteins were extracted from the both groups. The qPCR results showed that lncRNA GAS5 overexpression decreased the mRNA expression levels of fibrosis markers FN, Col-4, and TGFβ1 (Figure 8E–8G). Moreover, lncRNA GAS5 overexpression reduced the protein levels of FN, Col-4, and TGFβ1 in vivo (Figure 8H, 8I). To confirm the relationship between lncRNA GAS5 and miR-221 or SIRT1, the qPCR and western blot was performed in the DN tissues. Our finding indicated that lncRNA GAS5 overexpression downregulated the miR-221 expression and upregulated SIRT1 expression (Figure 8J–8M). Finally, the mRNA and protein expression level of p53 was detected in the DN tissues. Results indicated that lncRNA GAS5 overexpression increased the expression level of p53 (Figure 8N–8P). These data indicated that lncRNA GAS5 overexpression regulated miR-221 and SIRT1 expression and alleviated DN progression in vivo. Additionally, the expression levels of lncRNA GAS5 and SIRT1 were detected in T2D with DN tissues (N=30). Result revealed that there is positively correlation between lncRNA GAS5 and SIRT1 (Figure 8Q).

## DISCUSSION

The aim of this study is to investigate the expression pattern and biological function of lncRNA GAS5 in DN. Our finding revealed that the lncRNA GAS5 expression was downregulated in DN and is negatively associated with the severity of DN-related complications. Then, the DN rat model was established by injection with STZ. The qPCR results showed that the expression level of lncRNA GAS5 expression decreased in the DN rat model. The effect of lncRNA GAS5 on proliferation and fibrosis was investigated. lncRNA GAS5 overexpression significantly inhibited cell proliferation and fibrosis in the MCs. Conversely, the silencing of lncRNA GAS5 contributed to cell proliferation and fibrosis. An increasing number of studies have demonstrated that the lncRNA-miRNA-gene network is a central regulation mechanism in post-transcriptional gene regulation. lncRNA SNHG6 downregulates EZH2 expression by acting as a sponge for miR-26a/b and miR-214 in colorectal cancer [20]. lncRNA PVT1-214 enhances cell invasion and proliferation by sponging miR-128 in colorectal cancer [19]. lncRNA NEAT1 inhibits cell invasion by targeting miR-132 to modulate SOX2 expression in glioma cells [21]. To serve as an effective miRNA sponge, lncRNAs should be mainly localized in the cytoplasm [22]. Thus, the nuclear and cytoplasmic RNAs in the MCs were isolated. The qPCR and FISH assay results showed that lncRNA GAS5 was mainly expressed in the cytoplasm, suggesting that lncRNA GAS5 functions as a regulator through ceRNA mechanisms.

Previous studies have demonstrated that miRNAs are vital regulators in the development of DN. miR-23b alleviates albuminuria and fibrosis by targeting G3BP2 in DN [23]. In DN rat model, miR-370 regulates cell extracellular matrix and proliferation by targeting canopy 1 [24]. MiR-133b and miR-199b inhibition suppresses TGFβ1-induced EMT and renal fibrosis by targeting SIRT1 in DN [25]. MiR-221 was predicted as a potential target of lncRNA GAS5 (https://diana.e-ce.uth.gr/lncbasev3). We performed luciferase reporter assay to confirm the binding relationship between lncRNA GAS5 and miR-221. The results showed that lncRNA GAS5 binds to miR-221 in a sequence-specific manner. miRNAs act as part of an RISC binds mRNA and lead to the regulation of gene expression [26]. The RIP assay revealed that lncRNA GAS5 functioned as an endogenous sponge for miR-221 in the MCs in an Ago2-dependent manner. We performed CCK8 and western blot assays to investigate the biological function of miR-221 on the MCs. The results showed that miR-221 inhibition downregulated the expression of fibrosis-related proteins and suppressed the proliferation of MCs. SIRT1 was predicted as a potential target of miR-221. SIRT1 is involved in the progression of DN [27, 28]. The results showed that miR-221 binds to SIRT1 in a sequence-specific manner. Moreover, miR-221 inhibited proliferation and fibrosis-related proteins by targeting SIRT1. The relationship between lncRNA GAS5 and SIRT1 was further studied. The qPCR and western blot results revealed that the expression levels of lncRNA GAS5 and SIRT1 are positively correlated in the MCs. The rescuing experiment results showed that lncRNA GAS5 upregulated SIRT1 expression and suppressed cell proliferation and fibrosis-related proteins by acting as an miR-221 sponge. Finally, overexpression lncRNA GAS5 rat models were established by injection with LV-GAS5. Our finding revealed that lncRNA GAS5 overexpression significantly inhibited the development of DN in vivo. Specifically, fibrosis-related proteins FN, Col-4, and TGFβ1 were downregulated, and p53 was upregulated in the LV-GAS5 group. Additionally, lncRNA GAS5 significantly increased SIRT1 expression and downregulated miR-221 expression level in vivo.

Thus, we identified lncRNA GAS5 as an important negative regulator in DN progression. lncRNA GAS5 inhibited cell proliferation and fibrosis in DN by sponging miR-221 and modulating SIRT1 expression. This lncRNA GAS5/miR-221/SIRT1 network may shed light on the development of DN and may contribute to therapeutic targeting for DN.

## MATERIALS AND METHODS

### Cell culture

Mesangial cells (RAW264.7) were maintained in RPMI1640 (Gibco, USA) supplemented with 20% FBS (Gibco, USA) in an incubator (37 °C, 5% CO2). Mesangial cells were cultured at different glucose concentrations: 5.5 mM glucose (NC) and 30 mM glucose (high glucose).

### Flow cytometry analysis

Mesangial cells were transfected for 48 h and fixed in precooled ethanol at 4 °C overnight. The mesangial cells were washed with precooled PBS and stained in PI/Triton X-100, which contain 20μg of PI and 0.1% Triton X-100 to which 0.2 mg of RNase A was added. Flow cytometry was performed by a standard procedure. Three independent flow cytometry analyses were performed.

### Cell proliferation assay

CCK-8 and EDU assays were used to assess cell proliferation. In the CCK8 assay, mesangial cells were transfected for 36 h and plated at a 96-well plate containing 100 μl DMEM medium. Then, 10uM CCK-8 regent (Dingguo BioTechnology, China) was added. After 2h incubation at 37 °C, the microplate reader was utilized at 450nm. In the EDU assays, mesangial cells were transfected for 36h and plated at a 96-well plate. Then, 50μM EdU (RiboBio, China) was added into the plate. After the 2h incubation at 37 °C, the cells were fixed with 4% paraformaldehyde. According to standard procedures, anti-EdU working solution and Hoechst 33342 were utilized to stain and incubate the cells. Three independent experiments were performed.

### Reverse transcription PCR and quantitative real-time PCR

Total RNA was extracted by using TRIzol Reagent (Invitrogen, US). The expression levels of the mRNAs were assessed by using an SYBR Premix ExTaqTM II (Takara, China). FN, TGFβ1, TNF-α, and GAPDH expression levels were detected by using the following specific primers: F-5′-CGGTGGCTGTCAGTCAAAG-3′ and R-5′-AAACCTCGGCTTCCTCCATAA-3′; F-5′-GGCCAGATCCTGTCCAAGC-3′ and R-5′-GTGGGTT TCCACCATTAGCAC-3′; F-5′-CCTCTCTCTAATCAG CCCTCTG-3′ and R-5′-GAGGACCTGGGAGTAGATG AG-3′; F-5′-GGAGCGAGATCCCTCCAAAAT-3′ and R-5′-GGCTGTTGTCATACTTCTCATGG-3′. The two primers of GAS5 were as follows: forward, 5′-CGCG GATCCGTGCTGGGTGCAGATGCAGTGTGgc-3′ and reverse, 5′-CCGCTCGAGTTTTTTTTTTTTTTTTTTTT TTT-3′.

### Fluorescence in site hybridization

Fluorescence in site hybridization was done according to the literature [15]. Mesangial cells were permeabilized with precooled Triton X-100 (0.1%), and the slides were treated with paraformaldehyde (4%). Then, hybridization was performed with the lncRNA GSA5 probe for a night. The cells were washed with SCC buffer. DAPI was used to dye the coverslip, and laser scanning confocal microscope was utilized to test fluorescence.

### RNA extraction, lentivirus production, and cell transfection

TRIzol Reagent (Life USA) was utilized in RNA isolation. RNA samples with Abs260nm/Abs280nm ratio of >1.8 were used. Lentivirus vector for lncRNA GSA5 was built by Shanghai Genechem Co (China). miRNA-mimic/miRNA-inhibitor and corresponding controls were bought from RiboBio (China). The sequence of SIRT1 was subcloned into the plasmid vector (pcDNA3.1). The empty vector acted as negative control. The SIRT1 siRNA sequences were sense 5′-CCCUGUAAAGCUUUCAGAAdtdt-3′ and antisense 5′-UUCUGAAAGCUUUACAGGGdtdt-3′ (Genepharma, Shanghai, China). Lipofectamine 2000 (Life USA) was used for transfection in accordance with the manufacturer’s instructions.

### Nuclear and cytoplasmic RNA extraction

Nuclear and Cytoplasmic RNA Purification Kit (Norgen, USA) was used for the extraction and separation of cytoplasm and nuclear RNA. The isolated RNA was detected by qPCR.

### Dual-luciferase reporter assay

Dual-luciferase reporter assay was performed according to the literature [29]. A segment of the lncRNA GAS5 region contains the predicted binding site for miR-221 based on the LncBase (https://diana.e-ce.uth.gr/lncbasev3). The WT or MUT lncRNA GAS5 binding site was subcloned into pCDNA3.1 plasmid. The 293 T line was plated in the 24-well plates 24 h before transfection. miR-221-mimic, miR-221-inhibitor, and corresponding control were co-transfected with 10μg of pCDNA3.1-WT-GAS5 or pCDNA3.1-MUT-GAS5. Luciferase activity was detected with a dual-luciferase reporter assay system (Promega).

### RNA immunoprecipitation assays

RIP assay was carried out according to the literature [30] and standard protocols. The RIP immunoprecipitation buffer contained magnetic beads conjugated with IgG (control), and the Ago2 antibody (Millipore) was used to incubate the cell lysate. Magna RIP kit (EMD Millipore USA) was used in the RIP experiments. The coprecipitated RNAs were detected by qPCR.

### Western blot

From each sample, 16–20 mg of protein was extracted and utilized for Western blot. The antibodies utilized in this research included anti-FN (1:1000; Abcam; HK), anti-Col-4 (1:1000; Abcam; HK), and anti-TGFβ1 (1:1000; Abcam; HK). GAPDH (1:2000; Abcam; HK) was utilized as the loading control.

### Immunofluorescence assay

Triton X-100(0.1%) was used to permeate the cells, and goat serum solution (5%) was used to block the fixed cells. Primary antibodies were added on the coverslips for a night at 4 °C. Then, the coverslips were treated with Cy3-labeled secondary antibody for 1h. Finally, the coverslips were treated with DAPI and sealed by glycerine.

### Bioinformatics analysis

A segment of the lncRNA GAS5 region contains the predicted binding site for miR-221 based on the LncBase (https://diana.e-ce.uth.gr/lncbasev3). The target genes of miR-221 were predicted by miRNA.org (http://www.microrna.org/microrna/home.do).

### Animal models

Animal models were handled according to the literature [20]. Male Sprague-Dawley rats (8 weeks, n=8 for every group) were bought from Shanghai Laboratory Animal Center (Shanghai, China). STZ (50 mg/kg) was used to establish DN rat models. STZ was dissolved in 0.1M citrate acid buffer. Then, a DN rate model was established by the intraperitoneal injection of STZ once a day for 5 days. Blood glucose levels of more than 16.5 mmol/L were identified as successful DN rate models. According to the experimental objective, DN rats were divided into two groups: DN + LV-NC and DN + LV-GAS5. For the detection of lncRNA GAS5 overexpression, the lentivirus vector for lncRNA GAS5 was used. LV- GAS5 or LV-NC (200ul) was injected into the DN rat models through the caudal vein. All the DN rate models were killed at the end of the 3^rd^ week. This study was approved by the ethic committee of Tongren Hospital Affiliated to Shanghai Jiaotong University and was based on the Guide for the Care and Use of Laboratory Animals of the NIH.

### Statistical analysis

Students’ t-test and one-way ANOVA by GraphPad Prism 5.0 software and SPSS 13.0 were used to perform the statistical analyses. Statistical data were expressed as mean ± standard deviation (SD). Differences were considered significant at P<0.05.

## References

[1] Giacco F, Brownlee M. Oxidative stress and diabetic complications. Circ Res. 2010; 107:1058–70. 10.1161/CIRCRESAHA.110.223545 21030723 PMC2996922

[2] Araki E, Nishikawa T. Oxidative stress: A cause and therapeutic target of diabetic complications. J Diabetes Investig. 2010; 1:90–96. 10.1111/j.2040-1124.2010.00013.x 24843413 PMC4008021

[3] Wu H, Cai L, de Haan JB, Giacconi R. Targeting Oxidative Stress in Diabetic Complications: new Insights. J Diabetes Res. 2018; 2018:1909675. 10.1155/2018/1909675 30018984 PMC6029497

[4] Pickering RJ, Rosado CJ, Sharma A, Buksh S, Tate M, de Haan JB. Recent novel approaches to limit oxidative stress and inflammation in diabetic complications. Clin Transl Immunology. 2018; 7:e1016. 10.1002/cti2.1016 29713471 PMC5905388

[5] Mohsen L, Akmal DM, Ghonaim EK, Riad NM. Role of mean platelet volume and ischemia modified albumin in evaluation of oxidative stress and its association with postnatal complications in infants of diabetic mothers. J Matern Fetal Neonatal Med. 2018; 31:1819–23. 10.1080/14767058.2017.1330329 28502205

[6] Hu Q, Li C, Wang S, Li Y, Wen B, Zhang Y, Liang K, Yao J, Ye Y, Hsiao H, Nguyen TK, Park PK, Egranov SD, et al. LncRNAs-directed PTEN enzymatic switch governs epithelial-mesenchymal transition. Cell Res. 2019; 29:286–304. 10.1038/s41422-018-0134-3 30631154 PMC6461864

[7] Li Q, Pang L, Yang W, Liu X, Su G, Dong Y. Long Non-Coding RNA of Myocardial Infarction Associated Transcript (LncRNA-MIAT) Promotes Diabetic Retinopathy by Upregulating Transforming Growth Factor-β1 (TGF-β1) Signaling. Med Sci Monit. 2018; 24:9497–503. 10.12659/MSM.911787 30595603 PMC6328291

[8] Shan TD, Lv SY, Tian ZB, Liu XS, Liu FG, Sun XG. Knockdown of lncRNA H19 inhibits abnormal differentiation of small intestinal epithelial cells in diabetic mice. J Cell Physiol. 2018; 234:837–48. 10.1002/jcp.26902 30078183

[9] Xu X, Tian J, Li QY. Downregulation of HOTTIP regulates insulin secretion and cell cycle in islet β cells via inhibiting MEK/ERK pathway. Eur Rev Med Pharmacol Sci. 2018; 22:4962–68. 10.26355/eurrev_201808_15636 30070332

[10] Carter G, Miladinovic B, Patel AA, Deland L, Mastorides S, Patel NA. Circulating long noncoding RNA GAS5 levels are correlated to prevalence of type 2 diabetes mellitus. BBA Clin. 2015; 4:102–07. 10.1016/j.bbacli.2015.09.001 26675493 PMC4661729

[11] Zeng B, Li Y, Jiang F, Wei C, Chen G, Zhang W, Zhao W, Yu D. LncRNA GAS5 suppresses proliferation, migration, invasion, and epithelial-mesenchymal transition in oral squamous cell carcinoma by regulating the miR-21/PTEN axis. Exp Cell Res. 2019; 374:365–73. 10.1016/j.yexcr.2018.12.014 30576678

[12] Zhao H, Yu H, Zheng J, Ning N, Tang F, Yang Y, Wang Y. Lowly-expressed lncRNA GAS5 facilitates progression of ovarian cancer through targeting miR-196-5p and thereby regulating HOXA5. Gynecol Oncol. 2018; 151:345–55. 10.1016/j.ygyno.2018.08.032 30201235

[13] Iqbal MA, Arora S, Prakasam G, Calin GA, Syed MA. MicroRNA in lung cancer: role, mechanisms, pathways and therapeutic relevance. Mol Aspects Med. 2018. [Epub ahead of print]. 10.1016/j.mam.2018.07.003 30102929

[14] Gao J, Wang W, Wang F, Guo C. LncRNA-NR_033515 promotes proliferation, fibrogenesis and epithelial-to-mesenchymal transition by targeting miR-743b-5p in diabetic nephropathy. Biomed Pharmacother. 2018; 106:543–52. 10.1016/j.biopha.2018.06.104 29990842

[15] Li A, Peng R, Sun Y, Liu H, Peng H, Zhang Z. LincRNA 1700020I14Rik alleviates cell proliferation and fibrosis in diabetic nephropathy via miR-34a-5p/Sirt1/HIF-1α signaling. Cell Death Dis. 2018; 9:461. 10.1038/s41419-018-0527-8 29700282 PMC5919933

[16] Bai X, Geng J, Li X, Wan J, Liu J, Zhou Z, Liu X. Long Noncoding RNA LINC01619 Regulates MicroRNA-27a/Forkhead Box Protein O1 and Endoplasmic Reticulum Stress-Mediated Podocyte Injury in Diabetic Nephropathy. Antioxid Redox Signal. 2018; 29:355–76. 10.1089/ars.2017.7278 29334763

[17] Liu HN, Li X, Wu N, Tong MM, Chen S, Zhu SS, Qian W, Chen XL. Serum microRNA-221 as a biomarker for diabetic retinopathy in patients associated with type 2 diabetes. Int J Ophthalmol. 2018; 11:1889–94. 10.18240/ijo.2018.12.02 30588418 PMC6288522

[18] Lightell DJ Jr, Moss SC, Woods TC. Upregulation of miR-221 and -222 in response to increased extracellular signal-regulated kinases ½ activity exacerbates neointimal hyperplasia in diabetes mellitus. Atherosclerosis. 2018; 269:71–78. 10.1016/j.atherosclerosis.2017.12.016 29276985 PMC5812823

[19] He F, Song Z, Chen H, Chen Z, Yang P, Li W, Yang Z, Zhang T, Wang F, Wei J, Wei F, Wang Q, Cao J. Long noncoding RNA PVT1-214 promotes proliferation and invasion of colorectal cancer by stabilizing Lin28 and interacting with miR-128. Oncogene. 2019; 38:164–79. 10.1038/s41388-018-0432-8 30076414 PMC6329639

[20] Xu M, Chen X, Lin K, Zeng K, Liu X, Xu X, Pan B, Xu T, Sun L, He B, Pan Y, Sun H, Wang S. lncRNA SNHG6 regulates EZH2 expression by sponging miR-26a/b and miR-214 in colorectal cancer. J Hematol Oncol. 2019; 12:3. 10.1186/s13045-018-0690-5 30626446 PMC6327409

[21] Zhou K, Zhang C, Yao H, Zhang X, Zhou Y, Che Y, Huang Y. Knockdown of long non-coding RNA NEAT1 inhibits glioma cell migration and invasion via modulation of SOX2 targeted by miR-132. Mol Cancer. 2018; 17:105. 10.1186/s12943-018-0849-2 30053878 PMC6064054

[22] Dong H, Hu J, Zou K, Ye M, Chen Y, Wu C, Chen X, Han M. Activation of LncRNA TINCR by H3K27 acetylation promotes Trastuzumab resistance and epithelial-mesenchymal transition by targeting MicroRNA-125b in breast Cancer. Mol Cancer. 2019; 18:3. 10.1186/s12943-018-0931-9 30621694 PMC6323810

[23] Zhao B, Li H, Liu J, Han P, Zhang C, Bai H, Yuan X, Wang X, Li L, Ma H, Jin X, Chu Y. MicroRNA-23b Targets Ras GTPase-Activating Protein SH3 Domain-Binding Protein 2 to Alleviate Fibrosis and Albuminuria in Diabetic Nephropathy. J Am Soc Nephrol. 2016; 27:2597–608. 10.1681/ASN.2015030300 26839366 PMC5004638

[24] Yu FN, Hu ML, Wang XF, Li XP, Zhang BH, Lu XQ, Wang RQ. Effects of microRNA-370 on mesangial cell proliferation and extracellular matrix accumulation by binding to canopy 1 in a rat model of diabetic nephropathy. J Cell Physiol. 2019; 234:6898–6907. 10.1002/jcp.27448 30317577

[25] Sun Z, Ma Y, Chen F, Wang S, Chen B, Shi J. miR-133b and miR-199b knockdown attenuate TGF-β1-induced epithelial to mesenchymal transition and renal fibrosis by targeting SIRT1 in diabetic nephropathy. Eur J Pharmacol. 2018; 837:96–104. 10.1016/j.ejphar.2018.08.022 30125566

[26] Calvopina DA, Coleman MA, Lewindon PJ, Ramm GA. Function and Regulation of MicroRNAs and Their Potential as Biomarkers in Paediatric Liver Disease. Int J Mol Sci. 2016; 17:1795. 10.3390/ijms17111795 27801781 PMC5133796

[27] Liu R, Zhong Y, Li X, Chen H, Jim B, Zhou MM, Chuang PY, He JC. Role of transcription factor acetylation in diabetic kidney disease. Diabetes. 2014; 63:2440–53. 10.2337/db13-1810 24608443 PMC4066331

[28] Bible E. Diabetic nephropathy: Sirt1 attenuates diabetic albuminuria. Nat Rev Nephrol. 2013; 9:696. 10.1038/nrneph.2013.228 24166142

[29] Huang T, Yin L, Wu J, Gu JJ, Wu JZ, Chen D, Yu HL, Ding K, Zhang N, Du MY, Qian LX, Lu ZW, He X. MicroRNA-19b-3p regulates nasopharyngeal carcinoma radiosensitivity by targeting TNFAIP3/NF-κB axis. J Exp Clin Cancer Res. 2016; 35:188. 10.1186/s13046-016-0465-1 27919278 PMC5139034

[30] Wang X, Xu Y, Zhu YC, Wang YK, Li J, Li XY, Ji T, Bai SJ. LncRNA NEAT1 promotes extracellular matrix accumulation and epithelial-to-mesenchymal transition by targeting miR-27b-3p and ZEB1 in diabetic nephropathy. J Cell Physiol. 2019; 234:12926–12933. 10.1002/jcp.27959 30549040 PMC13483209

